# Population-Based Evaluation of Vaccine Effectiveness against SARS-CoV-2 Infection, Severe Illness, and Death, Taiwan

**DOI:** 10.3201/eid3003.230893

**Published:** 2024-03

**Authors:** Cheng-Yi Lee, Hung-Wei Kuo, Yu-Lun Liu, Jen-Hsiang Chuang, Jih-Haw Chou

**Affiliations:** Author affiliations: Taiwan Centers for Disease Control, Taipei, Taiwan (C.-Y. Lee, H.-W. Kuo, Y.-L. Liu, J.-H. Chuang); Ministry of Health and Welfare, Taipei (J.-H. Chou)

**Keywords:** COVID-19, respiratory infections, severe acute respiratory syndrome coronavirus 2, SARS-CoV-2, SARS, coronavirus disease, zoonoses, viruses, coronavirus, vaccine effectiveness, mix-and-match, population-based cohort study, AZD1222, mRNA-1273, BNT162b2, MVC-COV1901, Taiwan

## Abstract

Taiwan provided several COVID-19 vaccine platforms: mRNA (BNT162b2, mRNA-1273), adenoviral vector-based (AZD1222), and protein subunit (MVC-COV1901). After Taiwan shifted from its zero-COVID strategy in April 2022, population-based evaluation of vaccine effectiveness (VE) became possible. We conducted an observational cohort study of 21,416,151 persons to examine VE against SARS-CoV-2 infection, moderate and severe illness, and death during March 22, 2021–September 30, 2022. After adjusting for age and sex, we found that persons who completed 3 vaccine doses (2 primary, 1 booster) or received MVC-COV1901 as the primary series had the lowest hospitalization incidence (0.04–0.20 cases/100,000 person-days). We also found 95.8% VE against hospitalization for 3 doses of BNT162b2, 91.0% for MVC-COV1901, 81.8% for mRNA-1273, and 65.7% for AZD1222, which had the lowest overall VE. Our findings indicated that protein subunit vaccines provide similar protection against SARS-CoV-2­­–associated hospitalization as mRNA vaccines and can inform mix-and-match vaccine selection in other countries.

In response to the worldwide COVID-19 pandemic, most countries adopted vaccination policies on the basis of clinical trial outcomes and scientific evidence for vaccine procurement and policy planning frameworks. Studies suggested that after Omicron variants emerged, persons receiving 2 COVID-19 vaccine doses might not be adequately protected against severe illness and death (*1*–*8*). Research indicated that persons who completed a primary vaccine series would need a booster dose for better protection against new SARS-CoV-2 variants (*9*–*13*). Moreover, many countries provided several COVID-19 vaccine platform combinations (mix-and-match) of mRNA, protein subunit, and viral vector–based vaccines. However, few studies adopted population-level datasets and national vaccination registry records to examine the VE of mix-and-match COVID-19 vaccine regimens against SARS-CoV-2 infection, severe illness, and death.

Government agencies, including the UK Health Security Agency (*14*), the US Centers for Disease Control and Prevention (*7*), Health Canada (*15*), and the Public Health Agency of Sweden (*16*), adopted sampling or regional data to routinely evaluate COVID-19 VE in real-world settings. Those authorities review VE for national vaccination strategies to improve public policy implementation and provide evidence to encourage vulnerable groups and at-risk populations to get vaccinated. However, most countries worldwide have experienced several waves of the COVID-19 pandemic, and VE results could be affected by natural humoral immunity due to SARS-CoV-2 infection among populations. Thus, previous VE might be biased because of persons who were infected and vaccinated, reporting schemes, and fundamental distinctions among groups with different vaccination statuses.

Taiwan offers various COVID-19 vaccines for the public, including mRNA (Pfizer-BioNTech BNT162b2 [https://www.pfizer.com] and Moderna mRNA-1273 [https://www.modernatx.com]), protein subunit (Medigen MVC-COV1901 [https://www.medigenvac.com]), and Novavax NVX-CoV2373 [https://www.novavax.com]), and viral vector-based vaccines (Oxford–AstraZeneca AZD1222 [https://www.astrazeneca.com]). In Taiwan, AZD1222 was introduced on March 22, 2021, mRNA-1273 on June 8, 2021, MVC-COV1901 on August 23, 2021, and BNT162b2 on September 22, 2021. Government-funded COVID-19 vaccines were provided and prioritized by risk groups, such as healthcare workers, COVID-19 control staff (e.g., frontline health authority, customs, immigration, and quarantine staff, and security workers), caregivers in social welfare facilities, and high-risk groups (such as persons receiving kidney dialysis, older adults, pregnant women, and patients with rare diseases, catastrophic illnesses, or chronic diseases). No preferential recommendations for specific vaccine platforms were offered, and COVID-19 vaccines were provided to risk groups on the basis of availability. Persons could choose and reserve any available COVID-19 vaccine platforms at the vaccination stations.

In Taiwan, after authorities investigated COVID-19 cases, most were classified as imported, and few autochthonous cases were reported until April 2022. Community outbreaks did not begin until May 2021 and all were controlled within 3 months (*17*,*18*). Moreover, the national COVID-19 vaccination program was initiated in March 2021 (*19*), and vaccine coverage was <1% of the population when community outbreaks occurred in May 2021. Those outbreaks were mainly an Alpha subvariant of SARS-CoV-2 and was well controlled under the country’s zero-COVID policy. The Taiwan Centers for Disease Control (Taiwan CDC) conducted a seroprevalence survey on blood donors whose samples were obtained during January–April 2022. The national nucleocapsid protein positivity rate was 0.00%­–0.94%, showing that the population maintained a low level of COVID-19 infection. When a major outbreak of the SARS-CoV-2 Omicron BA.2 variant began in April 2022, the population could be regarded as SARS-CoV-2 immune naive. Thus, evaluating the nationwide VE of COVID-19 vaccines and vaccine combinations among a population-based cohort became realistic after April 2022.

We launched this study and used national vaccination registration records and a mandatory patient-level COVID-19 reporting dataset to estimate real-world VE of mRNA, protein subunit, and viral vector-based vaccines against infection, severe disease, and death in this predominantly infection-naive population during Omicron BA.2 variant predominance in Taiwan, mainly April–September 2022. This study also aimed to provide an overview and review of the performance of various COVID-19 vaccine platforms and vaccine combinations against the SARS-CoV-2–associated severe illness and to provide evidence for the vaccination strategy and guidance for areas and countries where various vaccine types are available.

## Methods

### Ethics Considerations

Taiwan CDC performed this study as a public policy analysis and evaluation. According to the Communicable Disease Control Act, Personal Data Protection Act, and regulations issued by the Ministry of Health and Welfare (reference no. 1010265083), the requirement of informed consent was waived from the study subjects because data were collected and obtained from Taiwan CDC. This study was approved by the Taiwan CDC institutional review board for health policy analysis research (reference no. 112103) and received an exempt review certificate of approval.

### Study Design and Data Sources

We conducted a population-based retrospective cohort study to assess the VE of mRNA (BNT162b2 and mRNA-1273), protein subunit (MVC-COV1901), and vector-based (ChAdOx1-S -AZD1222) COVID-19 vaccines in Taiwan during March 22, 2021–September 30, 2022. Our analysis included citizens and permanent residents of Taiwan. 

Registration in the National Immunization Information System (NIIS) is mandatory for all vaccinated persons and includes patient-level records of each government-funded vaccine administered. We retrieved the official database of the NIIS, which included vaccine types, vaccination dates of each dose, and vaccine combinations (i.e., mix-and-match) statuses for all vaccinees. We obtained information on SARS-CoV-2 infection notifications, moderate and severe illness (i.e., hospitalization), and death outcomes from the National Infectious Disease Reporting System (NIDRS). NIDRS also included information on eligible persons who were not vaccinated (i.e., received zero doses). At enrollment, NIIS and NDRS collected demographic information, such as age and sex, and information on enrollees’ residential districts. On November 10, 2022, we retrieved analytic datasets from Taiwan CDC systems that stored integrated data that integrated NIIS, NIDRS, and case information. To ensure that persons were alive at the start of the cohort, we verified personal identification numbers against the death registry and census database from the Ministry of the Interior.

The second booster (i.e., fourth dose) campaign for certain older adults and vulnerable groups began on May 16, 2022. Because persons who had 2 booster doses might have stronger immunity, we excluded persons whose records showed they had received a fourth dose (i.e., second booster) to avoid any possible bias. In addition to MVC-COV1901, Novavax (Nuvaxovid) is also a protein subunit vaccine. However, Novavax had limited availability and only specific population groups were eligible to receive it, so most persons could not receive Novavax in their primary vaccine series; thus, we excluded persons vaccinated with Novavax. Most COVID-19 case notifications occurred during April–September 2022, but the bivalent Moderna vaccine was not provided until September 2022; therefore, we excluded persons who received the Moderna bivalent vaccine. Of note, VE comparison of monovalent and bivalent vaccines was not the main goal of the study. 

### Statistical Analysis

Although the COVID-19 vaccine program launched on March 22, 2021, and most cases occurred after April 2022, sporadic outbreaks and community transmission still occurred and were attributed to imported cases during the zero-COVID strategy timeframe. Therefore, we estimated the overall VE of COVID-19 vaccines and aimed to provided VE of various mix-and-match vaccine platforms in Taiwan during March 22, 2021–September 30, 2022. Moreover, to address the timeframe between vaccination dates and events, we estimated the incidence rate and explored time from vaccination to infection, hospitalization, or death. We removed the total follow-up days and at-risk population if the outcome of interest occurred. We also explored incidence rates of outcomes of interest (i.e., confirmed infection, hospitalization, and death) for comparison. We considered persons protected at 14 days after a vaccine dose, the time required to develop an immune response. We calculated the person-days between the date of vaccination and event dates for infection, hospitalization, or death (Appendix).

We used logistic regression models to calculate odds ratios (ORs) and 95% CIs of hospitalization and death outcomes. We included vaccination status in the analysis and considered the demographic characteristics of sex and age as covariates. We defined VE as (1 – adjusted OR) × 100% to estimate the risk probability of outcomes of interest among persons who had 0, 1, 2, or 3 vaccine doses. We compared VEs of persons who had 1­–3 vaccination doses to unvaccinated (i.e., 0 vaccines) persons as the reference group. We also measured the absolute VE of various vaccine combinations against an unvaccinated reference (control) group. We excluded persons who received >4 vaccine doses from the analysis to avoid bias.

We stratified VE estimates by 3 age groups, all ages, 18–64 years of age, and >65 years of age, and by vaccine platform combinations (i.e., mix-and-match). Taiwan CDC guidelines did not restrict the brand of vaccines used as the primary series and encouraged eligible groups to receive vaccines when products were available. After a physician’s consultation, persons could choose their vaccine type and brand (Appendix). Because the array of combinations was infinite, to reduce confusion, we limited our analysis to 27 specific vaccine combinations, determined by the number of persons vaccinated. We performed all analyses in SAS version 9.4 (SAS Institute, Inc., https://www.sas.com) and SPSS Statistics 26.0 (IBM, https://www.ibm.com).

## Results

Our analysis included 23,933,482 unique persons, from which 2,516,382 persons were excluded because they received >4 vaccine doses during the study period; 949 persons were excluded because of incomplete national immunization and reporting system records. We found that 3,373,548 (15.8%) persons were unvaccinated, 1,183,138 (5.5%) received 1 dose, 3,287,659 (15.4%) received 2 doses, and 13,571,806 (63.4%) completed 3 doses (Table). The mean age was 41.0 years for unvaccinated persons, 28.7 years for persons with 1 vaccine dose, 31.8 years for persons with 2 doses, and 42.5 years for persons with 3 doses.

**Table:** Vaccination status and outcomes in a population-based evaluation of vaccine effectiveness against SARS-CoV-2 infection, severe illness, and death, Taiwan

Vaccination status and outcomes	Total population	Unvaccinated	No. doses		
			1	2	3
No. (%) cases	21,416,151 (100)	3,373,548 (15.8)	1,183,138 (5.5)	3,287,659 (15.4)	13,571,806 (63.4)
Mean age (SD)	39.9 (21.5)	41.0 (30.6)	28.7 (25.7)	31.8 (22.4)	42.5 (16.8)
Sex					
M	10,644,720	1,720,573	644,365	1,741,738	6,538,044
F	10,771,431	1,652,975	538,773	1,545,921	7,033,762
SARS-CoV-2 infection					
No. confirmed cases (%)	5,830,809 (27.2)	819,991 (24.3)	371,202 (31.4)	903,475 (27.5)	3,736,141 (27.5)
COVID-19 prognosis					
No. moderate and severe cases (%)	28,840 (0.13)	14,674 (0.43)	2,575 (0.22)	3,700 (0.11)	7,891 (0.06)
No. deaths (%)	10,667 (0.05)	5,342 (0.16)	989 (0.08)	1,305 (0.04)	3,031(0.02)

SARS-CoV-2 infection rates were 24.3% for unvaccinated (0 dose) persons, 31.4% for persons with 1 dose, 27.5% for persons with 2 doses, and 27.5% for persons with 3 doses. We found that 0.43% of unvaccinated persons had moderate to severe illness, which we defined by hospitalization, and 0.16% died. In contrast, 0.22% of 1-dose vaccinees were hospitalized and 0.08% died; 0.11% of 2-dose vaccinees were hospitalized and 0.04% died. Among persons who completed 3 doses, 0.06% were hospitalized and 0.02% died, which was the lowest death rate in our cohort.

We categorized 27 groups of vaccine combinations because of the complexity of mix-and-match combinations; we compiled the number of cases and patient characteristics and calculated the incidence of SARS-CoV-2 infection, hospitalization, and death (Appendix Table 1). Most persons who completed a 3-dose regimen received a combination of vaccines, most (3,769,921 [17.6%]) of which were 2 doses of AZD1222 and 1 dose of mRNA-1273.

For hospitalization risk comparison among 3-dose mix-and-match vaccine recipients, persons receiving MVC-COV1901 as the primary series had the lowest hospitalization incidence of 0.04–0.20/100,000 person-days, followed by BNT162b2 (0.06–0.20/100,000 person-days), mRNA-1273 (0.40–0.66/100,000 person-days), and AZD1222 (0.06–0.20/100,000 person-days). We observed a similar pattern in among patient deaths.

Among 3-dose vaccinees using the same brand, 3 doses of MVC-COV1901 had the lowest infection incidence (116.05 cases/100,000 person-days), followed by mRNA-1273 (138.11 cases/100,000 person-days), BNT162b2 (149.26 cases/100,000 person-days), and AZD1222 (152.62 cases/100,000 person-days). For COVID-19–associated hospitalization outcomes, 3 doses of BNT162b2 had the lowest incidence (0.06/100,000 person-days), followed by MVC-COV1901 (0.20/100,000 person-days), mRNA-1273 (0.48/100,000 person-days), and AZD1222 (0.71/100,000 person-days). We observed a similar pattern among patient deaths.

**Figure 1:**
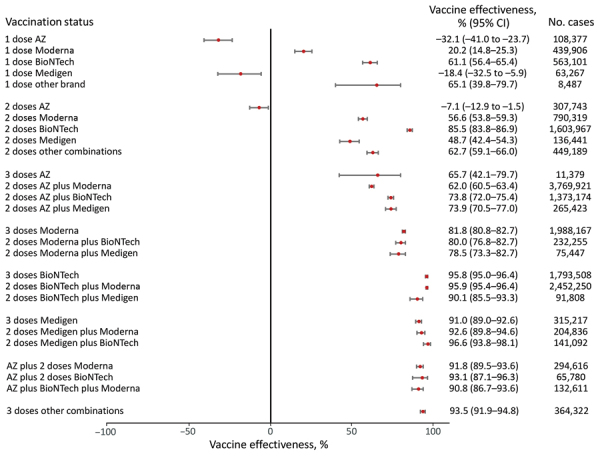
Vaccine effectiveness against hospitalization among all age groups in a population-based evaluation of vaccine effectiveness against SARS-CoV-2 infection, severe illness, and death, Taiwan, March 22, 2021–September 30, 2022. The study investigated various vaccine types: mRNA (Pfizer-BioNTech BNT162b2 [https://www.pfizer.com] and Moderna mRNA-1273 [https://www.modernatx.com]), protein subunit (Medigen MVC-COV1901 [https://www.medigenvac.com]), and viral vector–based vaccines (Oxford-AstraZeneca AZD1222 [https://www.astrazeneca.com]). The forest plot demonstrates effectiveness of different vaccination regimens status against moderate and severe illness defined by hospitalization for all age groups. Red dots indicate percentage effectiveness; bars indicate 95% CIs. AZ, AstraZeneca vaccine.

**Figure 2:**
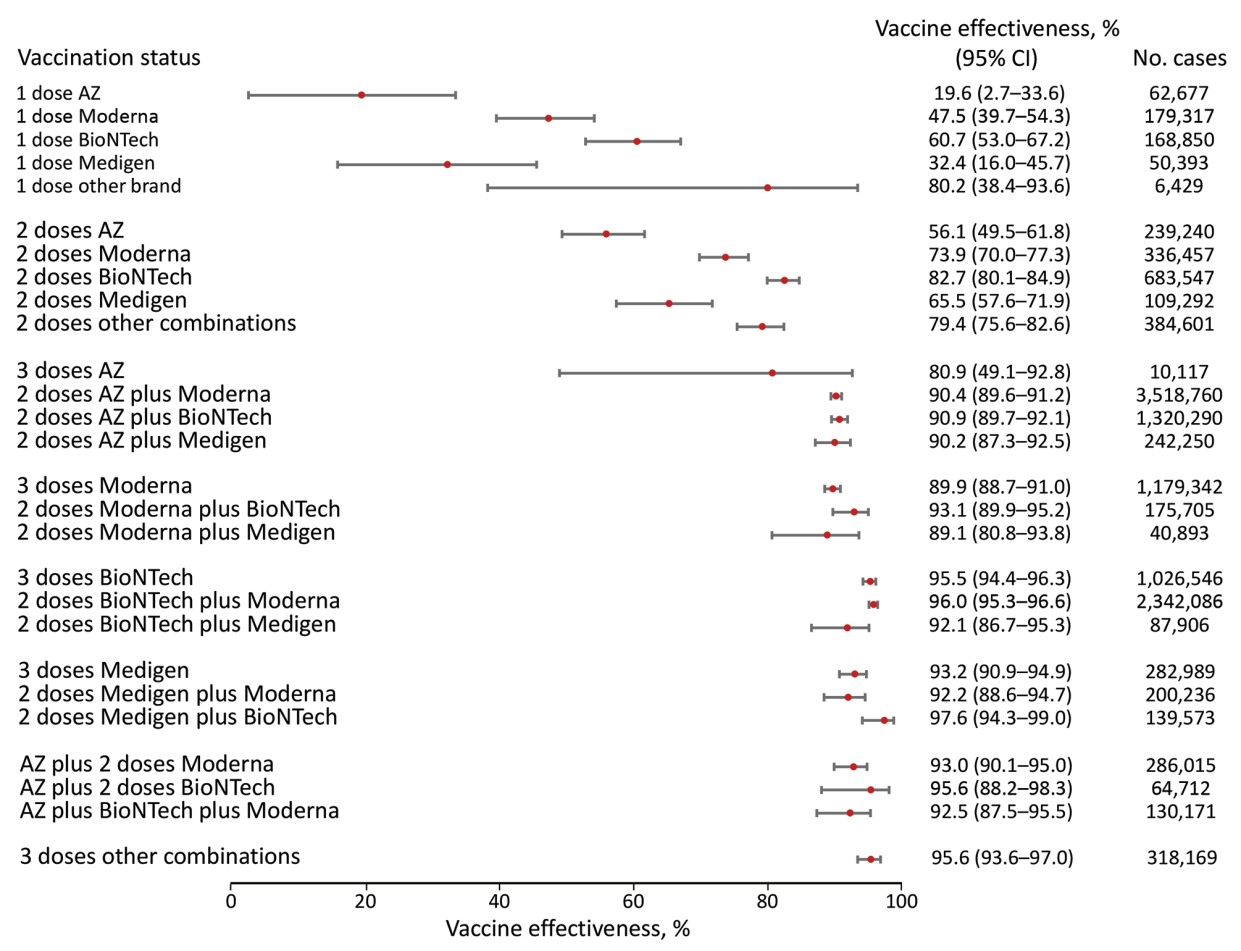
Vaccine effectiveness against hospitalization among persons 18–64 years of age in a population-based evaluation of vaccine effectiveness against SARS-CoV-2 infection, severe illness, and death, Taiwan, March 22, 2021–September 30, 2022. The study investigated various vaccine types: mRNA (Pfizer-BioNTech BNT162b2 [https://www.pfizer.com] and Moderna mRNA-1273 [https://www.modernatx.com]), protein subunit (Medigen MVC-COV1901 [https://www.medigenvac.com]), and viral vector–based vaccines (Oxford-AstraZeneca AZD1222 [https://www.astrazeneca.com]). The forest plot demonstrates effectiveness of different vaccination regimens status against moderate and severe illness defined by hospitalization for persons 18–64 years of age. Red dots indicate percentage effectiveness; bars indicate 95% CIs. AZ, AstraZeneca vaccine.

**Figure 3:**
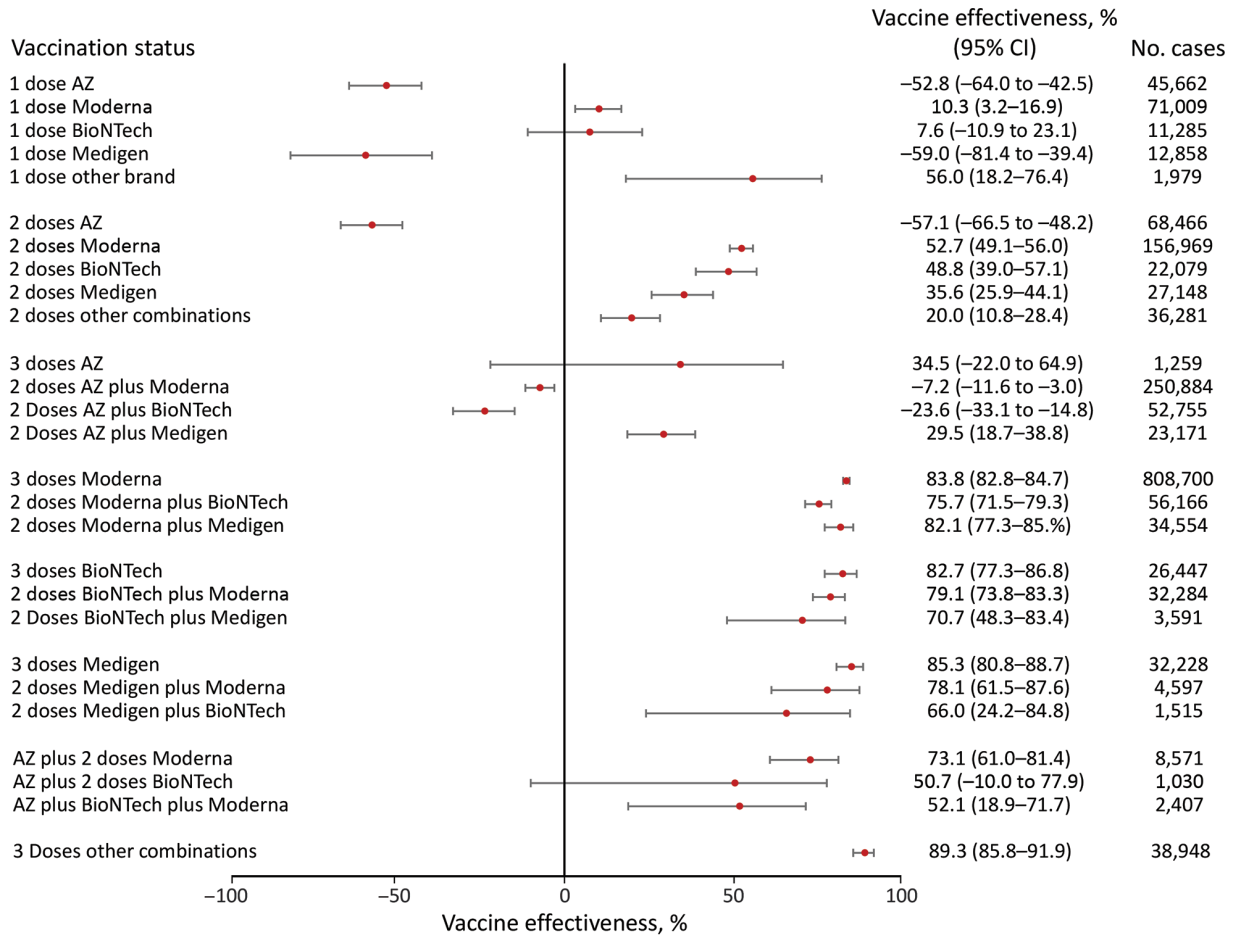
Vaccine effectiveness against hospitalization among persons >65 years of age in a population-based evaluation of vaccine effectiveness against SARS-CoV-2 infection, severe illness, and death, Taiwan, March 22, 2021–September 30, 2022. The study investigated various vaccine types: mRNA (Pfizer-BioNTech BNT162b2 [https://www.pfizer.com] and Moderna mRNA-1273 [https://www.modernatx.com]), protein subunit (Medigen MVC-COV1901 [https://www.medigenvac.com]), and viral vector-based vaccines (Oxford-AstraZeneca AZD1222 [https://www.astrazeneca.com]). The forest plot demonstrates effectiveness of different vaccination regimens status against moderate and severe illness defined by hospitalization for persons >65 years of age. Red dots indicate percentage effectiveness; bars indicate 95% CIs. AZ, AstraZeneca vaccine.

**Figure 4:**
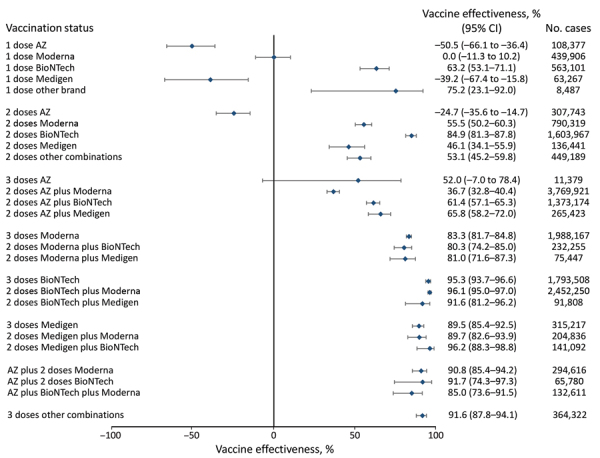
Vaccine effectiveness against death among all age groups in a population-based evaluation of vaccine effectiveness against SARS-CoV-2 infection, severe illness, and death, Taiwan, March 22, 2021–September 30, 2022. The study investigated various vaccine types: mRNA (Pfizer-BioNTech BNT162b2 [https://www.pfizer.com] and Moderna mRNA-1273 [https://www.modernatx.com]), protein subunit (Medigen MVC-COV1901 [https://www.medigenvac.com]), and viral vector-based vaccines (Oxford-AstraZeneca AZD1222 [https://www.astrazeneca.com]). The forest plot demonstrates effectiveness of different vaccination regimens status against death for all age groups. Blue diamonds indicate percentage effectiveness; bars indicate 95% CIs. AZ, AstraZeneca vaccine.

**Figure 5:**
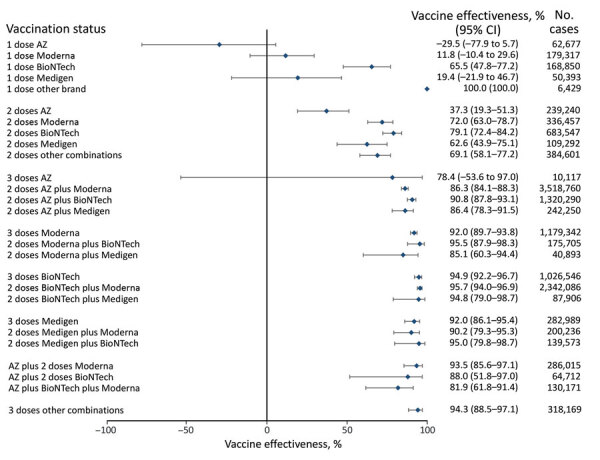
Vaccine effectiveness against death among persons 18–64 years of age in a population-based evaluation of vaccine effectiveness against SARS-CoV-2 infection, severe illness, and death, Taiwan, March 22, 2021–September 30, 2022. The study investigated various vaccine types: mRNA (Pfizer-BioNTech BNT162b2 [https://www.pfizer.com] and Moderna mRNA-1273 [https://www.modernatx.com protein subunit (Medigen MVC-COV1901 [https://www.medigenvac.com]), and viral vector–based vaccines (Oxford-AstraZeneca AZD1222 [https://www.astrazeneca.com]). The forest plot demonstrates effectiveness of different vaccination regimens status against death for persons 18–64 years of age. Blue diamonds indicate percentage effectiveness; bars indicate 95% CIs. AZ, AstraZeneca vaccine.

**Figure 6:**
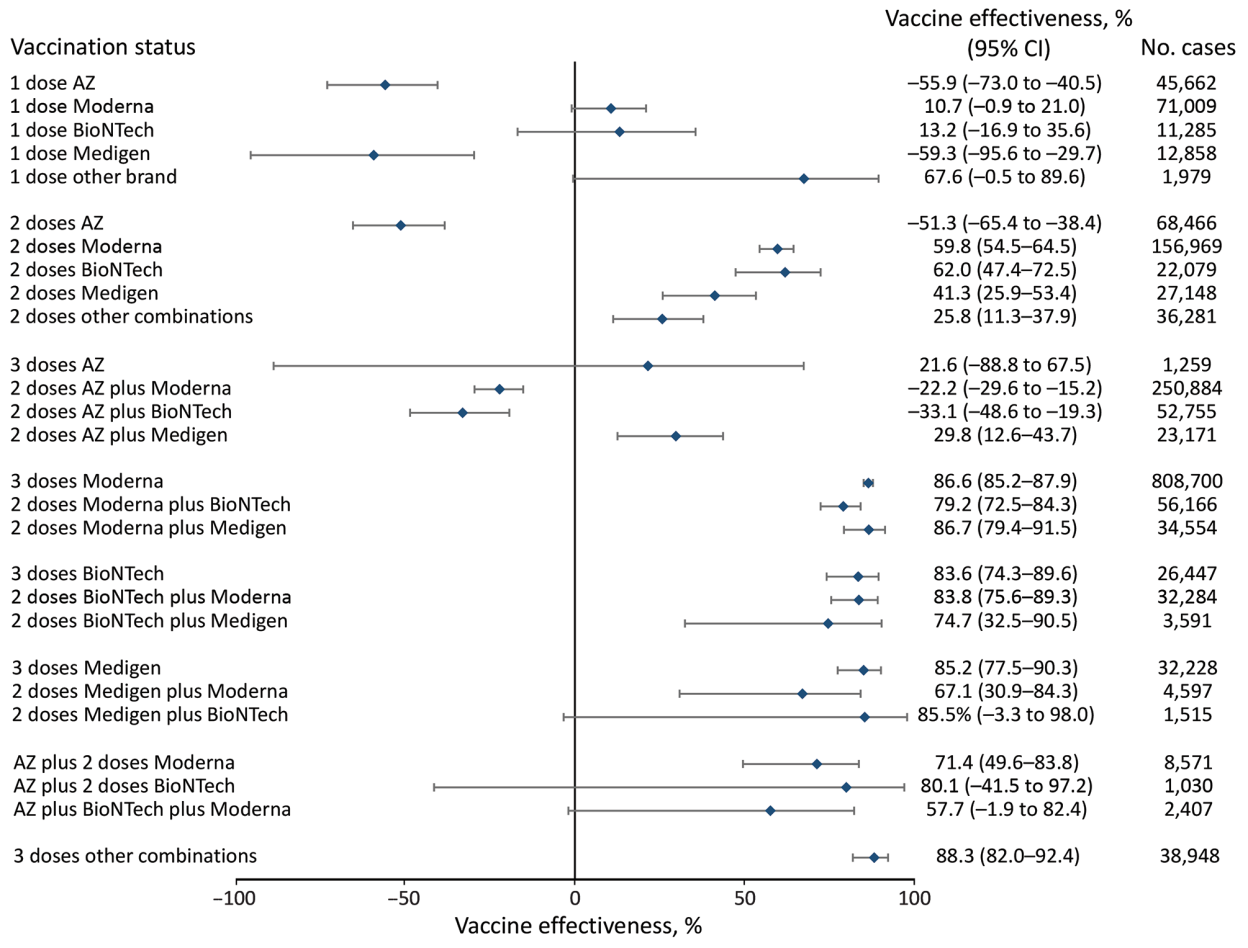
Vaccine effectiveness against death among persons >65 years of age in a population-based evaluation of vaccine effectiveness against SARS-CoV-2 infection, severe illness, and death, Taiwan, March 22, 2021–September 30, 2022. The study investigated various vaccine types: mRNA (Pfizer-BioNTech BNT162b2 [https://www.pfizer.com] and Moderna mRNA-1273 [https://www.modernatx.com]), protein subunit (Medigen MVC-COV1901 [https://www.medigenvac.com]), and viral vector–based vaccines (Oxford-AstraZeneca AZD1222 [https://www.astrazeneca.com]). The forest plot demonstrates effectiveness of different vaccination regimens status against death for persons >65 years of age. Blue diamonds indicate percentage effectiveness; bars indicate 95% CIs. AZ, AstraZeneca vaccine.

We categorized 3 age groups, all ages, 18­–64 years of age, and >65 years of age, to show VE against COVID-19–associated hospitalization and death (Appendix Tables 2, 3). We used unvaccinated persons as the reference group and adjusted for age and sex when calculating VE in multivariate models (Figures 1, 2, 3, 4, 5, 6). For VE against hospitalization, a booster dose generally provided higher protection (Figures 1, 2, 3). VE in persons who received mRNA vaccines as a primary series showed a similar pattern to persons who received protein-based vaccines as a primary series. We noted a 95.8% (95% CI 95.0%–96.4%) point estimate of VE for 3 doses of BNT162b2, an 81.8% (95% CI 80.8%–82.7%) point estimate for mRNA-1273, a 91.0% (95% CI 90.9%–92.6%) point estimate for MVC-COV1901, and a lower VE (65.7%; 95% CI 42.1%–79.9%) for 3 doses of AZD1222. In contrast, AZD1222 plus 2 doses of mRNA vaccines provided a higher (90.8%–93.1%) VE against hospitalization than 3 doses of AZD1222.

Among persons 18–64 years of age receiving only 1 dose, we observed no statistically significant protection against death for AZD1222 (−29.5%; 95% CI −77.9% to –5.7%), mRNA-1273 (11.8%; 95% CI −10.4% to 29.6%), BNT162b2 (65.5%; 95% CI 47.8%–77.2%), and MVC-COV1901 (19.4%; 95% CI –21.9% to 46.7%) (Figure 2). We observed a higher level of protection with 2 doses of mRNA; BNT162b2 reached 79.1% (95% CI 72.4%–84.25%) and mRNA-1273 reached 72.0% (95% CI 63.0%–78.7%). VE for protein-based vaccine MVC-COV1901 was 62.6% (95% CI 43.9%–75.1%). By comparison, the VE was the lowest, 37.3% (95% CI 19.3%–51.3%), for 3 AZD1222 doses.

We found higher VE was obtained among persons 18­–64 years of age who had a booster dose. In addition, 3 doses of mRNA or protein-based vaccines provided similar protection against COVID-19–associated death: 94.9% (95% CI 92.2%–96.7%) for BNT162b2, 92.0% (95% CI 89.7%–93.8%) for mRNA-1273, and 92.0% (95% CI 86.1%–95.4%) for MVC-COV1901 (Figure 2). The combination of 1 AZD1222 and 2 doses of mRNA vaccines provided high (81.9%–93.5%) protection, as well. However, we observed little protection from death after 3 doses of AZD1222, with a point estimate of 78.4% (95% CI −53.6% to 97.0%).

For persons >65 years of age who received 3 vaccine doses, 3 doses of mRNA or protein-based vaccines provided similar protection against death: 86.6% (95% CI 85.2%–87.9%) for mRNA-1273, 83.6% (95% CI 74.3%–89.6%) for BNT162b2, and 85.2% (95% CI 77.5%–90.3%) for MVC-COV1901 (Figure 6). However, 3 doses of AZD1222 provided low protection against death and had a point estimate of 21.6% (95% CI −88.8% to 67.5%). However, because of the relatively small population, we did not examine the combination of 1 AZD1222 and 2 doses of mRNA vaccines and other brands for this age group.

## Discussion

We adopted population-based data to evaluate effectiveness for different COVID-19 vaccine platforms among a predominately immune-naive population in Taiwan, which had minimal circulation of SARS-CoV-2 before March 2022. In April 2022, a major epidemic of Omicron BA.2 variant led the government to abandon its zero-COVID policy, which had been in effect since January 2020 (*20*). The nationwide vaccine campaign was initiated in March 2021. However, before the end of March 2022, the cumulative confirmed domestic cases were <0.3% of the total population. Therefore, evaluating nationwide COVID-19 vaccine effectiveness among this immune-naive population became possible in April 2022. According to nationwide community subvariant surveillance during April­ 2021–September 2022, the BA.2 Omicron SARS-CoV-2 variant predominated and accounted for 85%–90% of all subvariants; the rest were BA.5, BA.2.75, and others (*21*). Previous studies indicated that COVID-19 infection could induce natural immunity that can be as effective as vaccines for certain amount of time after infection (*22*–*25*). However, our study offers baseline immunity values of the effectiveness of various vaccine platform combinations, primarily induced by vaccines. In addition, our findings provide further data on vaccine-induced immunity against Omicron variants and VE of various mix-and-match vaccine platforms, rather than immunity from previous natural infection or a hybrid combination of protective effectiveness from vaccination and infection.

We found that persons who completed 3 vaccine doses and received mRNA platform vaccines (mRNA-1273 and BNT162b2) as the primary series had VE against COVID-19–associated hospitalization of 80.0%­–95.9%, and the VE against COVID-19–associated death was 80.3%–96.1%. For persons whose primary series doses were the protein subunit platform MVC-COV1901, VE against hospitalization was 91.0%–96.6%, and the VE against death was 89.5%–96.2%. For persons whose primary series doses were vector-based AZD1222, the VE against hospitalization was 62.0%–73.9%, and the VE against death was 36.7%–65.8%. The VE of mRNA and protein subunit vaccines against COVID-19 hospitalization and death were similar, but the VE of the vector-based vaccine was lower (Figures 1, 2, 3, 4, 5, 6).

A randomized, double-blind, active-controlled trial was conducted in Paraguay to evaluate immunogenicity of the protein subunit vaccine (*26*). Results from that study showed that MVC-COV1901 exhibited superiority in neutralizing antibody titers and noninferiority of seroconversion rates compared with the AZD1222 (*26*). A study on the protein recombinant vaccine NVX-CoV2373 (Novavax) in the general population of Italy found that VE against symptomatic COVID-19 was 31% (95% CI 16%–44%) in partially vaccinated (1 dose only) persons and 50% (95% CI 40%–58%) in fully vaccinated (2 doses) persons (*27*). Neither of those studies of protein vaccines reported VE against hospitalization and death. Our research might add insights and provide a reference for countries adopting MVC-COV1901 vaccines.

Our study provides additional information about VE among specific age groups and guidance for persons who might need second booster doses for better immunity, including persons whose primary series doses were AZD1222. For persons 18–64 years of age, our findings suggested that VE against COVID-19–associated hospitalization averaged ≈90%. However, VE against hospitalization and death for persons who received AZD1222 as primary series doses was lower than for those who received mRNA and protein subunit platform vaccines, suggesting vaccine-induced immunity waned more quickly for AZD1222 than for other vaccine types. That finding also might suggest that AZD1222 was not a proper choice for booster doses to induce sufficient immunity against SARS-CoV-2 Omicron variant, which is similar to a finding published by UK Health Security Agency (*28*). Our results indicated persons 18–64 years of age who completed 3 doses might have sufficient protection because VE against hospitalization was 80.9%–97.6% for that group, and the VE against death was 78.4%–95.7%. For persons >65 years of age, our findings indicated that persons whose primary series was AZD1222 had a VE against COVID-19­–associated hospitalization ranging from −23.6% to 34.5%, which was much lower than for persons receiving mRNA or subunit protein vaccine. Real-world data from Brazil showed similar results; among persons >60 years of age, VE against hospitalization for those receiving AZD1222 was lower than for those receiving mRNA platform, and waning immunity was reported (*29*). Future studies could explore whether persons receiving AZD1222 are at higher risk for waning immunity, hospitalization, and death compared with persons receiving other vaccine platforms.

The World Health Organization Strategic Advisory Group of Experts updated COVID-19 vaccination guidance in March 2023 (*30*). The advisory group indicated high-priority groups, which were mainly evaluated on the basis of risk for severe COVID-19and death. Our study suggested that the protection and immunity induced by vaccines among persons >65 years of age might not be sufficient, which is supported by previous studies in real-world settings (*28*,*31*,*32*). Therefore, the priority for future vaccine campaigns should emphasize persons >65 years of age, especially those whose primary series vaccines were AZD1222. The policy implication is that if a nationwide vaccine campaign was implemented with limited resources, the government could focus on the age groups and vaccine types that had lower VE rather than advocating for vaccination of the general population.

Our findings suggested that VE of the protein subunit vaccine MVC-COV1901 provides similar protection against COVID-19–associated hospitalization and death as mRNA vaccines BNT162b2 and mRNA-1273. Because both vaccine types could provide effective immunity against Omicron BA.2–associated severe outcomes, SARS-CoV-2 vaccine guidance in Taiwan recommend those vaccine types (*33*). VE of protein subunit and mRNA vaccines were also recognized by Indonesia, Palau, New Zealand, Belize, Somaliland, Thailand, Estonia, Paraguay, Malaysia, and Saint Kitts and Nevis (*33*). The similar VE of protein subunit and mRNA vaccines might provide the public with alternative vaccine types for primary series or booster shots. It also provides alternatives other than mRNA vaccines. 

We provide population-level VE evaluation of protein subunit vaccines against severe outcomes. However, other studies have reported the efficacy a similar vaccine, NVX-CoV2373 (Novavax), from clinical trials and VE against symptomatic infection (*27*,*34*). For public implications, the results from this study could enhance the autonomy of individual preferences. In addition, because the Medigen MVC-COV1901 vaccine was locally innovated and produced in Taiwan, fewer issues of availability might arise in the evolving pandemic.

The first limitation of this study is that in estimating VE, although we used age and sex for model adjustments, information on underlying conditions, medication, treatment status and history, health behaviors, and potential unmeasurable factors were unavailable for individual cases; thus, we could not include those confounding factors as variables. Second, because of variations in healthcare seeking behaviors, notification records might be underestimated, especially for mild or asymptomatic cases. Third, Taiwan CDC received hospitalization records from the clinical status, documentation, notification and investigation, and case reports from the NIIS and NIDRS rather than mandatory reporting; thus, moderate and severe illness (hospitalization) could have been underreported. Fourth, an expert committee reviewed each death case to verify whether the death was SARS-CoV-2–associated according to medical records and death certificates obtained by national cause of death registry. Therefore, the death case numbers might be underestimated compared with studies that defined SARS-CoV-2 death within a specific timeframe.

In summary, this study provides scientific evidence for countries that use several COVID-19 vaccine platform combinations (mix-and-match) of mRNA, protein subunit, and viral vector-based vaccines. The study identified which vaccine combinations have lower VE and might require additional booster shots or attention. We found that persons who received AZD1222 as their primary series vaccines might not be adequately protected against COVID-19–associated hospitalization and death, even if they received a booster dose. We also found that the protein subunit vaccine MVC-COV1901 provided similar protection against severe SARS-CoV-2 outcomes as mRNA vaccines. Our findings can help inform vaccine selection for various age groups and at-risk populations during future COVID-19 vaccination campaigns, especially if resources are limited. 

Appendix Additional information on a population-based evaluation of vaccine effectiveness against SARS-CoV-2 infection, severe illness, and death, Taiwan.
