# Supplementary material for: Population-Based Evaluation of Vaccine Effectiveness against SARS-CoV-2 Infection, Severe Illness, and Death, Taiwan
Source: Emerg Infect Dis. 2024 Mar;30(3):478–89. doi: 10.3201/eid3003.230893 (PMC10902541; doi:10.3201/eid3003.230893)
Supplement: Appendix — Additional information on a population-based evaluation of vaccine effectiveness against SARS-CoV-2 infection, severe illness, and death, Taiwan. [file 23-0893-Techapp-s1.pdf]

Article DOI: https://doi.org/10.3201/eid3003.230893
EID cannot ensure accessibility for supplementary materials supplied by authors. Readers who have difficulty accessing supplementary content should contact the authors for assistance.

Population-Based Evaluation of Vaccine Effectiveness against SARS-CoV-2 Infection, Severe Illness, and Death, Taiwan

Appendix

Definitions of Variables

Infection

Infection included symptomatic and asymptomatic cases. Individuals could visit the physician in the healthcare facility in person or via telemedicine under COVID-19 relevant regulations. The government provided free treatment for all SARS-CoV-2 confirmed cases regardless of nationality in Taiwan, which is under the regulation of Communicable Disease Act. The principle of the policy is to detect most cases and avoid community transmission, and therefore could also decrease the possibility of underreporting. The healthcare provider might perform a SARS-CoV-2 test with PCR or rapid antigen test; for telemedicine service, the individuals should provide positive proof of antigen tests to the clinicians. The clinicians would determine whether this individual was confirmed of infection by the validity of test results, symptoms, case contacts, travel history, and relevant clinical evidence.

After confirmation of infection, the physician must report each case and the essential diagnostic information to Taiwan CDC, which is mandatory under the Communicable Disease

Control Act. The definition and treatment guideline of reinfection was published in July 2022 by Taiwan CDC, and most cases were SARS-CoV-2 immune naive groups, we included the first infection (notification) record only and excluded the reinfection status or individuals who had known previous infection to avoid potential bias.

Moderate and Severe Illness (Hospitalization)

The definitions of moderate and severe cases, such as oxygen supplement, ICU admission, requiring extracorporeal membrane oxygenation (ECMO), acute respiratory distress syndrome (ARDS), or clinical status categorized as critical were followed by the World Health Organization case definition. Since some therapeutic drugs, including VEKLURY (Remdesivir), were distributed by regional health authorities. Taiwan CDC can collect those case details through the application forms and electronic medical records. In principle, Taiwan CDC can manage most of the status of moderate and severe cases. Moderate and severe illness (hospitalization) cases in the Taiwan CDC's definition include COVID-19 associated hospitalization and COVID-19 associated fatal (death) cases.

Deaths

In addition to the physician and health authority's fatal notification of COVID-19 confirmed cases, each case will be followed by an electronic dataset linkage with the death registry from the Department of Statistics, Ministry of Health and Welfare. This was to ensure the survival of the status of cases and could be guidance for public health policy implementation. Taiwan CDC's expert committee is reviewing each fatal case and determining whether the cause of death was associated with SARS-CoV-2.

Vaccination Status

Since the vaccination status was a dynamic variable over time, while each case was notified to the system, the symptom onset date (determined by the physician's or patient's statement), most recent vaccination date, number of doses, and vaccine types would be adopted for calculation. If the duration of the symptom onset date and the latest vaccination date were ≥14 days, this individual would be categorized as completed with the latest vaccination. However, if the day's duration were <14 days, e.g., 5 days after the second dose, the individual would be categorized as completed the previous dose vaccination since the individual may not be fully protected and developing the immune responses to vaccination until 7–14 days after this dose. We extracted data on all vaccination after the national COVID-19 vaccine campaign on March 22, 2021.

Appendix Table 1. Age characteristics and incidence of individuals receiving mRNA, protein subunit, vector-based and mix-and-match vaccines against SARS-CoV-2 infection and associated moderate and severe illness (hospitalization) and death*

		Mean age	Total follow-up	Confirmed infection		Moderate or severe illness‡		Death	
Characteristics, n = 21,416,151	No. cases (%)	(SD)	days†	No. cases	Incidence§	No. cases	Incidence§	No. deaths	Incidence§
Unvaccinated	3,373,548 (15.80)	41 (30.6)	1,787,702,880	819,991	45.87	14,674	0.82	5,342	0.30
One dose									
One dose AZ	108,377 (0.50)	59.2 (24.2)	41,784,112	21,572	51.63	976	2.34	432	1.03
One dose Moderna	439,906 (2.10)	31.4 (26.4)	68,243,076	131,195	192.25	967	1.42	369	0.54
One dose BioNTech	563,101 (2.60)	18 (18.2)	73,620,327	197,804	268.68	304	0.41	68	0.09
One dose Medigen	63,267 (0.30)	50.5 (16.8)	10,127,312	19,827	195.78	315	3.11	117	1.16
One dose other brand	8,487 (0.00)	51 (18.6)	611,391	804	131.50	13	2.13	3	0.49
Two doses									
Two doses AZ	307,743 (1.40)	49.6 (21.6)	90,422,409	98,177	108.58	1,519	0.17	617	0.07
Two doses Moderna	790,319 (3.70)	35.5 (26)	120,922,449	145,522	120.34	1,044	0.09	330	0.03
Two doses BioNTech	1,603,967 (7.50)	22.9 (17.5)	258,410,099	507,946	196.57	364	0.01	90	0.00
Two doses Medigen	136,441 (0.60)	50.6 (16.6)	23,794,896	32,433	136.30	294	0.12	97	0.04
Two doses other combinations	449,189 (2.10)	39.1 (17.2)	66,393,399	119,397	179.83	479	0.07	171	0.03
Three doses									
Three doses AZ	11,379 (0.10)	44.7 (17.4)	1,964,409	2,998	152.62	14	0.71	6	0.31
Two doses AZ plus Moderna	3,769,921 (17.60)	43.9 (15.3)	736,223,936	1,228,539	166.87	3,845	0.52	1,711	0.23
Two doses AZ plus BioNTech	1,373,174 (6.40)	42.9 (13.5)	285,212,457	443,395	155.46	1,008	0.35	398	0.14
Two doses AZ plus Medigen	265,423 (1.20)	47.8 (14.6)	49,023,479	72,214	147.30	252	0.51	99	0.20
Three doses Moderna	1,988,167 (9.30)	55.9 (16.2)	363,743,997	502,378	138.11	1,759	0.48	518	0.14
Two doses Moderna plus BioNTech	232,255 (1.10)	49.1 (16.8)	44,634,170	72,001	161.31	180	0.40	54	0.12

Characteristics, n = 21,416,151	No. cases (%)	Mean age (SD)	Total follow-up days†	Confirmed infection		Moderate or severe illness‡		Death	
				No. cases	Incidence§	No. cases	Incidence§	No. deaths	Incidence§
Two doses Moderna plus Medigen	75,447 (0.40)	57.3 (15.7)	12,513,966	18,647	149.01	83	0.66	24	0.19
Three doses BioNTech	1,793,508 (8.40)	30 (17)	257,651,017	384,558	149.26	150	0.06	41	0.02
Two doses BioNTech plus Moderna	2,452,250 (11.50)	38 (13.6)	385,601,220	631,180	163.69	244	0.06	60	0.02
Two doses BioNTech plus Medigen	91,808 (0.40)	41.9 (13.9)	12,894,890	22,349	173.32	26	0.20	6	0.05
Three doses Medigen	315,217 (1.50)	47.2 (14.5)	52,010,694	60,357	116.05	103	0.20	35	0.07
Two doses Medigen plus Moderna	204,836 (1.00)	38.7 (12.7)	37,990,293	54,635	143.81	39	0.10	14	0.04
Two doses Medigen plus BioNTech	141,092 (0.70)	36.5 (11.4)	29,357,624	43,405	147.85	11	0.04	3	0.01
AZ plus 2 doses Moderna	294,616 (1.40)	38.6 (13.3)	47,493,759	87,508	184.25	61	0.13	18	0.04
AZ plus 2 doses BioNTech	65,780 (0.30)	35.2 (12.7)	11,008,590	18,564	168.63	10	0.09	3	0.03
AZ plus BioNTech plus Moderna	132,611 (0.60)	36.1 (13.1)	19,646,067	36,747	187.05	29	0.15	12	0.06
Three doses other combinations	364,322 (1.70)	42.9 (16.5)	41,181,058	56,666	137.60	77	0.02	29	0.07

*AZ, Astra Zeneca; SD, standard deviation.

†Calculation of follow-up days, or person-days, was based on the time between the date of receiving vaccination and the date the event occurred. For unvaccinated persons, due to the lack of a date for receiving vaccination, the starting date was set as March 22, 2021, which was the beginning of the national COVID-19 vaccine campaign and study start date.

‡Hospitalization was considered severe or moderate illness.

§Incidence per 100,000 person-days. The incidence rate was used to compare risks for SARS-CoV-2–related infection, hospitalization, and death, which accounted for the follow-up days for vaccination types and the type of incident that occurred.

Appendix Table 2. Vaccine effectiveness of COVID-19 associated moderate and severe illness (hospitalization) by vaccine types and age groups*

Characteristics	No. (%)	Moderate and severe outcomes†			Vaccine effectiveness, % (95% CI)‡
		No. cases	Rate	Exp(β) (95% CI)	
All age groups, n = 21,416,151					
Unvaccinated	3,373,548 (15.75)	14,674	4.35	NA	NA
One dose					
One dose AZ	108,377 (0.51)	976	9.01	1.321 (1.237–1.410)	−32.1 (−41.0 to –23.7)
One dose Moderna	439,906 (2.05)	967	2.20	0.798 (0.747–0.852)	20.2 (14.8–25.3)
One dose BioNTech	563,101 (2.63)	304	0.54	0.389 (0.346–0.436)	61.1 (56.4–65.4)
One dose Medigen	63,267 (0.30)	315	4.98	1.184 (1.059–1.325)	−18.4 (−32.5 to 5.9)
One dose other brand	8,487 (0.04)	13	1.53	0.349 (0.203–0.602)	65.1 (39.8–79.7)
Two doses					
Two doses AZ	307,743 (1.44)	1,519	4.94	1.071 (1.015–1.129)	−7.1 (−12.9 to –1.5)
Two doses Moderna	790,319 (3.69)	1,044	1.32	0.434 (0.407–0.462)	56.6 (53.8–59.3)
Two doses BioNTech	1,603,967 (7.49)	364	0.23	0.145 (0.131–0.162)	85.5 (83.8–86.9)
Two doses Medigen	136,441 (0.64)	294	2.15	0.513 (0.457–0.576)	48.7 (42.4–54.3)
Two doses other combinations	449,189 (2.10)	479	1.07	0.373 (0.340–0.409)	62.7 (59.1–66.0)
Three doses					
Three doses AZ	11,379 (0.05)	14	1.23	0.343 (0.203–0.579)	65.7 (42.1–79.7)
Two doses AZ plus Moderna	3,769,921 (17.60)	3,845	1.02	0.380 (0.366–0.395)	62.0 (60.5–63.4)
Two doses AZ plus BioNTech	1,373,174 (6.41)	1,008	0.73	0.262 (0.246–0.280)	73.8 (72.0–75.4)
Two doses AZ plus Medigen	265,423 (1.24)	252	0.95	0.261 (0.230–0.295)	73.9 (70.5–77.0)
Three doses Moderna	1,988,167 (9.28)	1,759	0.88	0.182 (0.173–0.192)	81.8 (80.8–82.7)
Two doses Moderna plus BioNTech	232,255 (1.08)	180	0.78	0.200 (0.173–0.232)	80.0 (76.8–82.7)
Two doses Moderna plus Medigen	75,447 (0.35)	83	1.10	0.215 (0.173–0.267)	78.5 (73.3–82.7)
Three doses BioNTech	1,793,508 (8.37)	150	0.08	0.042 (0.036–0.050)	95.8 (95.0–96.4)
Two doses BioNTech plus Moderna	2,452,250 (11.45)	244	0.10	0.041 (0.036–0.046)	95.9 (95.4–96.4)
Two doses BioNTech plus Medigen	91,808 (0.43)	26	0.28	0.099 (0.067–0.145)	90.1 (85.5–93.3)
Three doses Medigen	315,217 (1.47)	103	0.33	0.090 (0.074–0.110)	91.0 (89.0–92.6)
Two doses Medigen plus Moderna	204,836 (0.96)	39	0.19	0.074 (0.054–0.102)	92.6 (89.8–94.6)
Two doses Medigen plus BioNTech	141,092 (0.66)	11	0.08	0.034 (0.019–0.062)	96.6 (93.8–98.1)
AZ plus 2 doses Moderna	294,616 (1.38)	61	0.21	0.082 (0.064–0.105)	91.8 (89.5–93.6)
AZ plus 2 doses BioNTech	65,780 (0.31)	10	0.15	0.069 (0.037–0.129)	93.1 (87.1–96.3)
AZ plus BioNTech plus Moderna	132,611 (0.62)	29	0.22	0.092 (0.064–0.133)	90.8 (86.7–93.6)

Characteristics	No. (%)	Moderate and severe outcomes†			Vaccine effectiveness, % (95% CI)‡
		No. cases	Rate	Exp(β) (95% CI)	
Three doses other combinations	364,322 (1.70)	77	0.21	0.065 (0.052–0.081)	93.5 (91.9–94.8)
18–64 y Age group, n = 15,008,667					
Unvaccinated	1,422,104 (9.48)	3,744	2.63	NA	NA
One dose					
One dose AZ	62,677 (0.42)	109	1.74	0.804 (0.664–0.973)	19.6 (2.7–33.6)
One dose Moderna	179,317 (1.19)	213	1.19	0.525 (0.457–0.603)	47.5 (39.7–54.3)
One dose BioNTech	168,850 (1.13)	124	0.73	0.393 (0.328–0.470)	60.7 (53.0–67.2)
One dose Medigen	50,393 (0.34)	83	1.65	0.676 (0.543–0.840)	32.4 (16.0–45.7)
One dose other brand	6,429 (0.04)	3	0.47	0.198 (0.064–0.616)	80.2 (38.4–93.6)
Two doses	0.00				
Two doses AZ	239,240 (1.59)	209	0.87	0.439 (0.382–0.505)	56.1 (49.5–61.8)
Two doses Moderna	336,457 (2.24)	209	0.62	0.261 (0.227–0.300)	73.9 (70.0–77.3)
Two doses BioNTech	683,547 (4.55)	211	0.31	0.173 (0.151–0.199)	82.7 (80.1–84.9)
Two doses Medigen	109,292 (0.73)	94	0.86	0.345 (0.281–0.424)	65.5 (57.6–71.9)
Two doses other combinations	384,601 (2.56)	140	0.36	0.206 (0.174–0.244)	79.4 (75.6–82.6)
Three doses	0.00				
Three doses AZ	10,117 (0.07)	4	0.40	0.191 (0.072–0.509)	80.9 (49.1–92.8)
Two doses AZ plus Moderna	3,518,760 (23.44)	676	0.19	0.096 (0.088–0.104)	90.4 (89.6–91.2)
Two doses AZ plus BioNTech	1,320,290 (8.80)	238	0.18	0.091 (0.079–0.103)	90.9 (89.7–92.1)
Two doses AZ plus Medigen	242,250 (1.61)	57	0.24	0.098 (0.075–0.127)	90.2 (87.3–92.5)
Three doses Moderna	1,179,342 (7.86)	323	0.27	0.101 (0.090–0.113)	89.9 (88.7–91.0)
Two doses Moderna plus BioNTech	175,705 (1.17)	27	0.15	0.069 (0.048–0.101)	93.1 (89.9–95.2)
Two doses Moderna plus Medigen	40,893 (0.27)	12	0.29	0.109 (0.062–0.192)	89.1 (80.8–93.8)
Three doses BioNTech	1,026,546 (6.84)	92	0.09	0.045 (0.037–0.056)	95.5 (94.4–96.3)
Two doses BioNTech plus Moderna	2,342,086 (15.60)	165	0.07	0.040 (0.034–0.047)	96.0 (95.3–96.6)
Two doses BioNTech plus Medigen	87,906 (0.59)	14	0.16	0.079 (0.047–0.133)	92.1 (86.7–95.3)
Three doses Medigen	282,989 (1.89)	47	0.17	0.068 (0.051–0.091)	93.2 (90.9–94.9)
Two doses Medigen plus Moderna	200,236 (1.33)	27	0.13	0.078 (0.053–0.114)	92.2 (88.6–94.7)
Two doses Medigen plus BioNTech	139,573 (0.93)	5	0.04	0.024 (0.010–0.057)	97.6 (94.3–99.0)
AZ plus 2 doses Moderna	286,015 (1.91)	33	0.12	0.070 (0.050–0.099)	93.0 (90.1–95.0)
AZ plus 2 doses BioNTech	64,712 (0.43)	4	0.06	0.044 (0.017–0.118)	95.6 (88.2–98.3)
AZ plus BioNTech plus Moderna	130,171 (0.87)	15	0.12	0.075 (0.045–0.125)	92.5 (87.5–95.5)
Three doses other combinations	318,169 (2.12)	28	0.09	0.044 (0.030–0.064)	95.6 (93.6–97.0)

Characteristics	No. (%)	Moderate and severe outcomes† No. cases	Rate	Exp(β) (95% CI)	Vaccine effectiveness, % (95% CI)‡
≥65 y Age group, n = 2,693,856					
Unvaccinated	861,013 (31.96)	10,328	NA	NA	NA
One dose					
One dose AZ	45,662 (1.70)	867	18.99	1.528 (1.425–1.640)	−52.8 (−64.0 to –42.5)
One dose Moderna	71,009 (2.64)	723	10.18	0.897 (0.831–0.968)	10.3 (3.2–16.9)
One dose BioNTech	11,285 (0.42)	118	10.46	0.924 (0.769–1.109)	7.6 (−10.9 to 23.1)
One dose Medigen	12,858 (0.48)	232	18.04	1.590 (1.394–1.814)	−59.0 (−81.4 to −39.4)
One dose other brand	1,979 (0.07)	10	5.05	0.440 (0.236–0.818)	56.0 (18.2–76.4)
Two doses					
Two doses AZ	68,466 (2.54)	1,310	19.13	1.571 (1.482–1.665)	−57.1 (−66.5 to –48.2)
Two doses Moderna	156,969 (5.83)	833	5.31	0.473 (0.440–0.509)	52.7 (49.1–56.0)
Two doses BioNTech	22,079 (0.82)	127	5.75	0.512 (0.429–0.610)	48.8 (39.0–57.1)
Two doses Medigen	27,148 (1.01)	200	7.37	0.644 (0.559–0.741)	35.6 (25.9–44.1)
Two doses other combinations	36,281 (1.35)	335	9.23	0.800 (0.716–0.892)	20.0 (10.8–28.4)
Three doses					
Three doses AZ	1,259 (0.05)	10	7.94	0.655 (0.351–1.220)	34.5 (22.0 to 64.9)
Two dose AZ plus Moderna	250,884 (9.31)	3,169	12.63	1.072 (1.030–1.116)	−7.2 (−11.6 to –3.0)
Two dose AZ plus BioNTech	52,755 (1.96)	770	14.60	1.236 (1.148–1.331)	−23.6 (−33.1 to –14.8)
Two dose AZ plus Medigen	23,171 (0.86)	195	8.42	0.705 (0.612–0.813)	29.5 (18.7–38.8)
Three doses Moderna	808,700 (30.02)	1,436	1.78	0.162 (0.153–0.172)	83.8 (82.8–84.7)
Two dose Moderna plus BioNTech	56,166 (2.08)	153	2.72	0.243 (0.207–0.285)	75.7 (71.5–79.3)
Two dose Moderna plus Medigen	34,554 (1.28)	71	2.05	0.179 (0.142–0.227)	82.1 (77.3–85.8)
Three doses BioNTech	26,447 (0.98)	52	1.97	0.173 (0.132–0.227)	82.7 (77.3–86.80)
Two dose BioNTech plus Moderna	32,284 (1.20)	77	2.39	0.209 (0.167–0.262)	79.1 (73.8–83.3)
Two dose BioNTech plus Medigen	3,591 (0.13)	12	3.34	0.293 (0.166–0.517)	70.7 (48.3–83.4)
Three doses Medigen	32,228 (1.20)	56	1.74	0.147 (0.113–0.192)	85.3 (80.8–88.7)
Two dose Medigen plus Moderna	4,597 (0.17)	12	2.61	0.219 (0.124–0.385)	78.1 (61.5–87.6)
Two dose Medigen plus BioNTech	1,515 (0.06)	6	3.96	0.340 (0.152–0.758)	66.0 (24.2–84.8)
AZ plus 2 doses Moderna	8,571 (0.32)	28	3.27	0.269 (0.186–0.390)	73.1 (61.0–81.4)
AZ plus 2 doses BioNTech	1,030 (0.04)	6	5.83	0.493 (0.221–1.100)	50.7 (−10.0 to 77.9)
AZ plus BioNTech plus Moderna	2,407 (0.09)	14	5.82	0.479 (0.283–0.811)	52.1 (18.9–71.7)
Three dose other combinations	38,948 (1.45)	49	1.26	0.107 (0.081–0.142)	89.3 (85.8–91.9)

*AZ, Astra Zeneca; NA, not applicable.

Characteristics	No. (%)	Moderate and severe outcomes† No. cases	Rate	Exp(β) (95% CI)	Vaccine effectiveness, % (95% CI)‡

†Adjusted for age and sex. Rate per 1,000 population.

‡Vaccine effectiveness = 1 – Exp(β)

Appendix Table 3. Vaccine effectiveness of COVID-19–associated death by vaccine types and age group*

		Death†			
Characteristics	No. (%)	No. cases	Mortality rate	Exp(β) (95% CI)	Vaccine effectiveness, % (95% CI)
All age groups, n = 21,416,151					
Unvaccinated	3,373,548 (15.75)	5,342	15.83	NA	NA
One dose					
One dose AZ	108,377 (0.51)	432	39.86	1.505 (1.364–1.661)	−50.5 (−66.1 to –36.4)
One dose Moderna	439,906 (2.05)	369	8.39	1.000 (0.898–1.113)	0.0 (−11.3 to 10.2)
One dose BioNTech	563,101 (2.63)	68	1.21	0.368 (0.289–0.469)	63.2 (53.1–71.1)
One dose Medigen	63,267 (0.30)	117	18.49	1.392 (1.158–1.674)	−39.2 (−67.4 to –15.8)
One dose other brand	8,487 (0.04)	3	3.53	0.248 (0.080–0.769)	75.2 (23.1–92.0)
Two doses					
Two doses AZ	307,743 (1.44)	617	20.05	1.247 (1.147–1.356)	−24.7 (−35.6 to –14.7)
Two doses Moderna	790,319 (3.69)	330	4.18	0.445 (0.397–0.498)	55.5 (50.2–60.3)
Two doses BioNTech	1,603,967 (7.49)	90	0.56	0.151 (0.122–0.187)	84.9 (81.3–87.8)
Two doses Medigen	136,441 (0.64)	97	7.11	0.539 (0.441–0.659)	46.1 (34.1–55.9)
Two doses other combinations	449,189 (2.10)	171	3.81	0.469 (0.402–0.548)	53.1 (45.2–59.8)
Three doses					
Three doses AZ	11,379 (0.05)	6	5.27	0.480 (0.216–1.070)	52.0 (−7.0 to 78.4)
Two doses AZ plus Moderna	3,769,921 (17.60)	1,711	4.54	0.633 (0.596–0.672)	36.7 (32.8–40.4)
Two doses AZ plus BioNTech	1,373,174 (6.41)	398	2.90	0.386 (0.347–0.429)	61.4 (57.1–65.3)
Two doses AZ plus Medigen	265,423 (1.24)	99	3.73	0.342 (0.280–0.418)	65.8 (58.2–72.0)
Three doses Moderna	1,988,167 (9.28)	518	2.61	0.167 (0.152–0.183)	83.3 (81.7–84.8)
Two doses Moderna plus BioNTech	232,255 (1.08)	54	2.33	0.197 (0.150–0.258)	80.3 (74.2–85.0)
Two doses Moderna plus Medigen	75,447 (0.35)	24	3.18	0.190 (0.127–0.284)	81.0 (71.6–87.3)
Three doses BioNTech	1,793,508 (8.37)	41	0.23	0.047 (0.034–0.063)	95.3 (93.7–96.6)

Characteristics	No. (%)	Death† No. cases	Mortality rate	Exp(β) (95% CI)	Vaccine effectiveness, % (95% CI)
Two doses BioNTech plus Moderna	2,452,250 (11.45)	60	0.24	0.039 (0.030–0.050)	96.1 (95.0–97.0)
Two doses BioNTech plus Medigen	91,808 (0.43)	6	0.65	0.084 (0.038–0.188)	91.6 (81.2–96.2)
Three doses Medigen	315,217 (1.47)	35	1.11	0.105 (0.075–0.146)	89.5 (85.4–92.5)
Two doses Medigen plus Moderna	204,836 (0.96)	14	0.68	0.103 (0.061–0.174)	89.7 (82.6–93.9)
Two doses Medigen plus BioNTech	141,092 (0.66)	3	0.21	0.038 (0.012–0.117)	96.2 (88.3–98.8)
AZ plus 2 doses Moderna	294,616 (1.38)	18	0.61	0.092 (0.058–0.146)	90.8 (85.4–94.2)
AZ plus 2 doses BioNTech	65,780 (0.31)	3	0.46	0.083 (0.027–0.257)	91.7 (74.3–97.3)
AZ plus BioNTech plus Moderna	132,611 (0.62)	12	0.90	0.150 (0.085–0.264)	85.0 (73.6–91.5)
Three doses other combinations	364,322 (1.70)	29	0.80	0.084 (0.059–0.122)	91.6 (87.8–94.1)
18–64 y age group, n = 15,008,667					
Unvaccinated	1,422,104 (9.48)	905	6.36	NA	NA
One dose					
One dose AZ	62,677 (0.42)	40	6.38	1.295 (0.943–1.779)	−29.5 (−77.9 to –5.7)
One dose Moderna	179,317 (1.19)	83	4.63	0.882 (0.704–1.104)	11.8 (−10.4 to 29.6)
One dose BioNTech	168,850 (1.13)	23	1.36	0.345 (0.228–0.522)	65.5 (47.8–77.2)
One dose Medigen	50,393 (0.34)	23	4.56	0.806 (0.533–1.219)	19.4 (−21.9 to 46.7)
One dose other brand	6,429 (0.04)	0	0.00	0.0	100.0 (100.0)
Two doses					
Two doses AZ	239,240 (1.59)	65	2.72	0.627 (0.487–0.807)	37.3 (19.3–51.3)
Two doses Moderna	336,457 (2.24)	53	1.58	0.280 (0.213–0.370)	72.0 (63.0–78.7)
Two doses BioNTech	683,547 (4.55)	53	0.78	0.209 (0.158–0.276)	79.1 (72.4–84.2)
Two doses Medigen	109,292 (0.73)	24	2.20	0.374 (0.249–0.561)	62.6 (43.9–75.1)
Two doses other combinations	384,601 (2.56)	44	1.14	0.309 (0.228–0.419)	69.1 (58.1–77.2)
Three doses					
Three doses AZ	10,117 (0.07)	1	0.99	0.216 (0.030–1.536)	78.4 (−53.6 to 97.0)
Two doses AZ plus Moderna	3,518,760 (23.44)	208	0.59	0.137 (0.117–0.159)	86.3 (84.1–88.3)
Two doses AZ plus BioNTech	1,320,290 (8.80)	51	0.39	0.092 (0.069–0.122)	90.8 (87.8–93.1)
Two doses AZ plus Medigen	242,250 (1.61)	18	0.74	0.136 (0.085–0.217)	86.4 (78.3–91.5)
Three doses Moderna	1,179,342 (7.86)	63	0.53	0.080 (0.062–0.103)	92.0 (89.7–93.8)

Characteristics	No. (%)	Death† No. cases	Mortality rate	Exp(β) (95% CI)	Vaccine effectiveness, % (95% CI)
Two doses Moderna plus BioNTech	175,705 (1.17)	4	0.23	0.045 (0.017–0.121)	95.5 (87.9–98.3)
Two doses Moderna plus Medigen	40,893 (0.27)	4	0.98	0.149 (0.056–0.397)	85.1 (60.3–94.4)
Three doses BioNTech	1,026,546 (6.84)	22	0.21	0.051 (0.033–0.078)	94.9 (92.2–96.7)
Two doses BioNTech plus Moderna	2,342,086 (15.60)	37	0.16	0.043 (0.031–0.060)	95.7 (94.0–96.9)
Two doses BioNTech plus Medigen	87,906 (0.59)	2	0.23	0.052 (0.013–0.210)	94.8 (79.0–98.7)
Three doses Medigen	282,989 (1.89)	13	0.46	0.080 (0.046–0.139)	92.0 (86.1–95.4)
Two doses Medigen plus Moderna	200,236 (1.33)	7	0.35	0.098 (0.047–0.207)	90.2 (79.3–95.3)
Two doses Medigen plus BioNTech	139,573 (0.93)	2	0.14	0.050 (0.013–0.202)	95.0 (79.8–98.7)
AZ plus 2 doses Moderna	286,015 (1.91)	6	0.21	0.065 (0.029–0.144)	93.5 (85.6–97.1)
AZ plus 2 doses BioNTech	64,712 (0.43)	2	0.31	0.120 (0.030–0.482)	88.0 (51.8–97.0)
AZ plus BioNTech plus Moderna	130,171 (0.87)	7	0.54	0.181 (0.086–0.382)	81.9 (61.8–91.4)
Three dose other combinations	318,169 (2.12)	8	0.25	0.057 (0.029–0.115)	94.3 (88.5–97.1)
≥65 y age group, n = 2,693,856					
Unvaccinated	861,013 (31.96)	4,402	51.13	NA	NA
One dose					
One dose AZ	45,662 (1.70)	392	85.85	1.559 (1.405–1.730)	−55.9 (−73.0 to –40.5)
One dose Moderna	71,009 (2.64)	284	39.99	0.893 (0.790–1.009)	10.7 (−0.9 to 21.0)
One dose BioNTech	11,285 (0.42)	44	38.99	0.868 (0.644–1.169)	13.2 (−16.9 to 35.6)
One dose Medigen	12,858 (0.48)	94	73.11	1.593 (1.297–1.956)	−59.3 (−95.6 to –29.7)
One dose other brand	1,979 (0.07)	3	15.16	0.324 (0.104–1.005)	67.6 (−0.5 to 89.6)
Two doses					
Two doses AZ	68,466 (2.54)	552	80.62	1.513 (1.384–1.654)	−51.3 (−65.4 to –38.4)
Two doses Moderna	156,969 (5.83)	277	17.65	0.402 (0.355–0.455)	59.8 (54.5–64.5)
Two doses BioNTech	22,079 (0.82)	37	16.76	0.380 (0.275–0.526)	62.0 (47.4–72.5)
Two doses Medigen	27,148 (1.01)	73	26.89	0.587 (0.466–0.741)	41.3 (25.9–53.4)
Two doses other combinations	36,281 (1.35)	126	34.73	0.742 (0.621–0.887)	25.8 (11.3–37.9)
Three doses					
Three doses AZ	1,259 (0.05)	5	39.71	0.784 (0.325–1.888)	21.6 (−88.8 to 67.5)

		Death†			
Characteristics	No. (%)	No. cases	Mortality rate	Exp(β) (95% CI)	Vaccine effectiveness, % (95% CI)
Two doses AZ plus Moderna	250,884 (9.31)	1,503	59.91	1.222 (1.152–1.296)	−22.2 (−29.6 to –15.2)
Two doses AZ plus BioNTech	52,755 (1.96)	347	65.78	1.331 (1.193–1.486)	−33.1 (−48.6 to –19.3)
Two doses AZ plus Medigen	23,171 (0.86)	81	34.96	0.702 (0.563–0.874)	29.8 (12.6–43.7)
Three doses Moderna	808,700 (30.02)	455	5.63	0.134 (0.121–0.148)	86.6 (85.2–87.9)
Two doses Moderna plus BioNTech	56,166 (2.08)	50	8.90	0.208 (0.157–0.275)	79.2 (72.5–84.3)
Two doses Moderna plus Medigen	34,554 (1.28)	20	5.79	0.133 (0.085–0.206)	86.7 (79.4–91.5)
Three doses BioNTech	26,447 (0.98)	19	7.18	0.164 (0.104–0.257)	83.6 (74.3–89.6)
Two doses BioNTech plus Moderna	32,284 (1.20)	23	7.12	0.162 (0.107–0.244)	83.8 (75.6–89.3)
Two doses BioNTech plus Medigen	3,591 (0.13)	4	11.14	0.253 (0.095–0.675)	74.7 (32.5–90.5)
Three doses Medigen	32,228 (1.20)	22	6.83	0.148 (0.097–0.225)	85.2 (77.5–90.3)
Two doses Medigen plus Moderna	4,597 (0.17)	7	15.23	0.329 (0.157–0.691)	67.1 (30.9–84.3)
Two doses Medigen plus BioNTech	1,515 (0.06)	1	6.60	0.145 (0.020–1.033)	85.5 (−3.3 to 98.0)
AZ plus 2 doses Moderna	8,571 (0.32)	12	14.00	0.286 (0.162–0.504)	71.4 (49.6–83.8)
AZ plus 2 doses BioNTech	1,030 (0.04)	1	9.71	0.199 (0.028–1.415)	80.1 (−41.5 to 97.2)
AZ plus BioNTech plus Moderna	2,407 (0.09)	5	20.77	0.423 (0.176–1.019)	57.7 (−1.9 to 82.4)
Three dose other combinations	38,948 (1.45)	21	5.39	0.117 (0.076–0.180)	88.3 (82.0–92.4)

*AZ, Astra Zeneca; NA, not applicable.

†Adjusted for age and sex. Rate per 10,000 population.

‡Vaccine effectiveness = 1 – Exp(β).

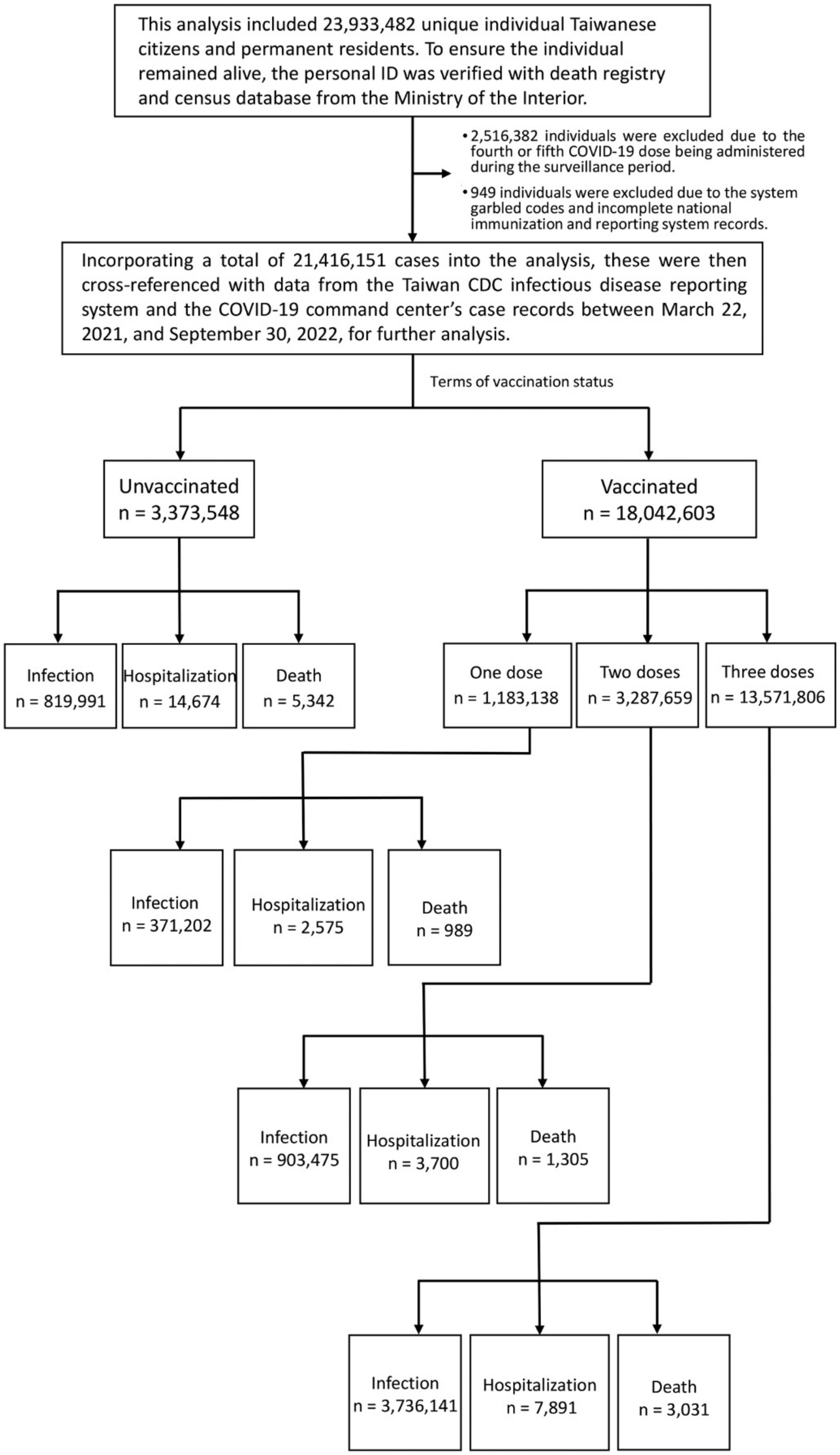

Appendix Figure. Flowchart of eligibility for subjects in a population-based evaluation of vaccine effectiveness against SARS-CoV-2 infection, severe illness, and death, Taiwan.
